# A Bayesian Developmental Approach to Robotic Goal-Based Imitation Learning

**DOI:** 10.1371/journal.pone.0141965

**Published:** 2015-11-04

**Authors:** Michael Jae-Yoon Chung, Abram L. Friesen, Dieter Fox, Andrew N. Meltzoff, Rajesh P. N. Rao

**Affiliations:** 1 Department of Computer Science & Engineering, University of Washington, Seattle, WA, United States of America; 2 Institute for Learning & Brain Sciences, University of Washington, Seattle, WA, United States of America; Centre for Coevolution of Biology & Culture, University of Durham, UNITED KINGDOM

## Abstract

A fundamental challenge in robotics today is building robots that can learn new skills by observing humans and imitating human actions. We propose a new Bayesian approach to robotic learning by imitation inspired by the developmental hypothesis that children use self-experience to bootstrap the process of intention recognition and goal-based imitation. Our approach allows an autonomous agent to: (i) learn probabilistic models of actions through self-discovery and experience, (ii) utilize these learned models for inferring the goals of human actions, and (iii) perform goal-based imitation for robotic learning and human-robot collaboration. Such an approach allows a robot to leverage its increasing repertoire of learned behaviors to interpret increasingly complex human actions and use the inferred goals for imitation, even when the robot has very different actuators from humans. We demonstrate our approach using two different scenarios: (i) a simulated robot that learns human-like gaze following behavior, and (ii) a robot that learns to imitate human actions in a tabletop organization task. In both cases, the agent learns a probabilistic model of its own actions, and uses this model for goal inference and goal-based imitation. We also show that the robotic agent can use its probabilistic model to seek human assistance when it recognizes that its inferred actions are too uncertain, risky, or impossible to perform, thereby opening the door to human-robot collaboration.

## Introduction

Considerable progress has been made in robotics in recent years, particularly in the area of human-robot interaction. Techniques have been proposed to impart new skills to robots via “programming by demonstration” [1] and “imitation learning” [2]. An important remaining challenge is endowing a robot with the ability to infer the intentions of humans and to learn new skills not by naively following demonstrated action trajectories (“trajectory-based” or “action” imitation) but through *goal-based imitation* [3, 4].

Developmental scientists have shown that infants can infer the goal of an adult’s actions. In one set of experiments with 18-month old infants [5], an adult actor demonstrated an act in which the goal-state was not achieved; infants were able to read through the literal body movements to infer the underlying goal and execute the intended act. In other experiments, children employed different means to achieve the same goal as adults. When given a barbell-shaped object, adults used their hands to pull apart the object, but children who could not grasp the end of an oversized barbell used alternative means (e.g., holding one end in both hands and clasping the other end between their knees) to pull it apart. The children thus not only inferred the adult’s goal but could also use novel alternative means to achieve the goal.

Clues to how children acquire the ability to infer goals have come from studies on gaze following, the ability of humans to follow the line of regard of another human. A naive interpretation of gaze following is that it is simply the imitation of head movement. However, Meltzoff and Brooks [6] controlled for head movements and showed that infants’ tendency to follow gaze varied as a function of whether or not the actor’s view was blocked by an intervening opaque occluder (a blindfold). In particular, 18-month-old children did not follow the gaze of an adult who made a head movement toward an object while wearing a blindfold. Younger children (12-month-olds) did mistakenly follow the gaze of a blindfolded adult. However, after these younger children were given the self-experience of a blindfold blocking their own view, they no longer followed the gaze of the blindfolded adult.

These results highlight the importance of self-experience in the development of goal inference capabilities and goal-based imitation. They are consistent with Meltzoff’s “Like-Me” hypothesis [7] which states that children utilize internal models learned through self-experience to interpret the acts and intentions of others, and with increasing experience, acquire increasingly sophisticated intent recognition abilities. Such an approach is different from but incorporates elements of two previous well-known theories of intent inference: (i) “simulation theory” [8], which proposes that the same mental resources we use for thinking, decision-making, and emotional responses are redeployed in imagination to provide an understanding of others, and (ii) “theory theory” [9, 10], which advocates that children develop theories about the world and about others, make predictive inferences about behaviors and inner states of others using this network of beliefs (“theories”), and revise theories according to new observations. The approach taken here differs by providing a rigorous mathematical framework based on Bayesian inference of goals and actions, and the bootstrapping of Bayesian models through learning from self-experience.

## Methods

**Fig 1:**
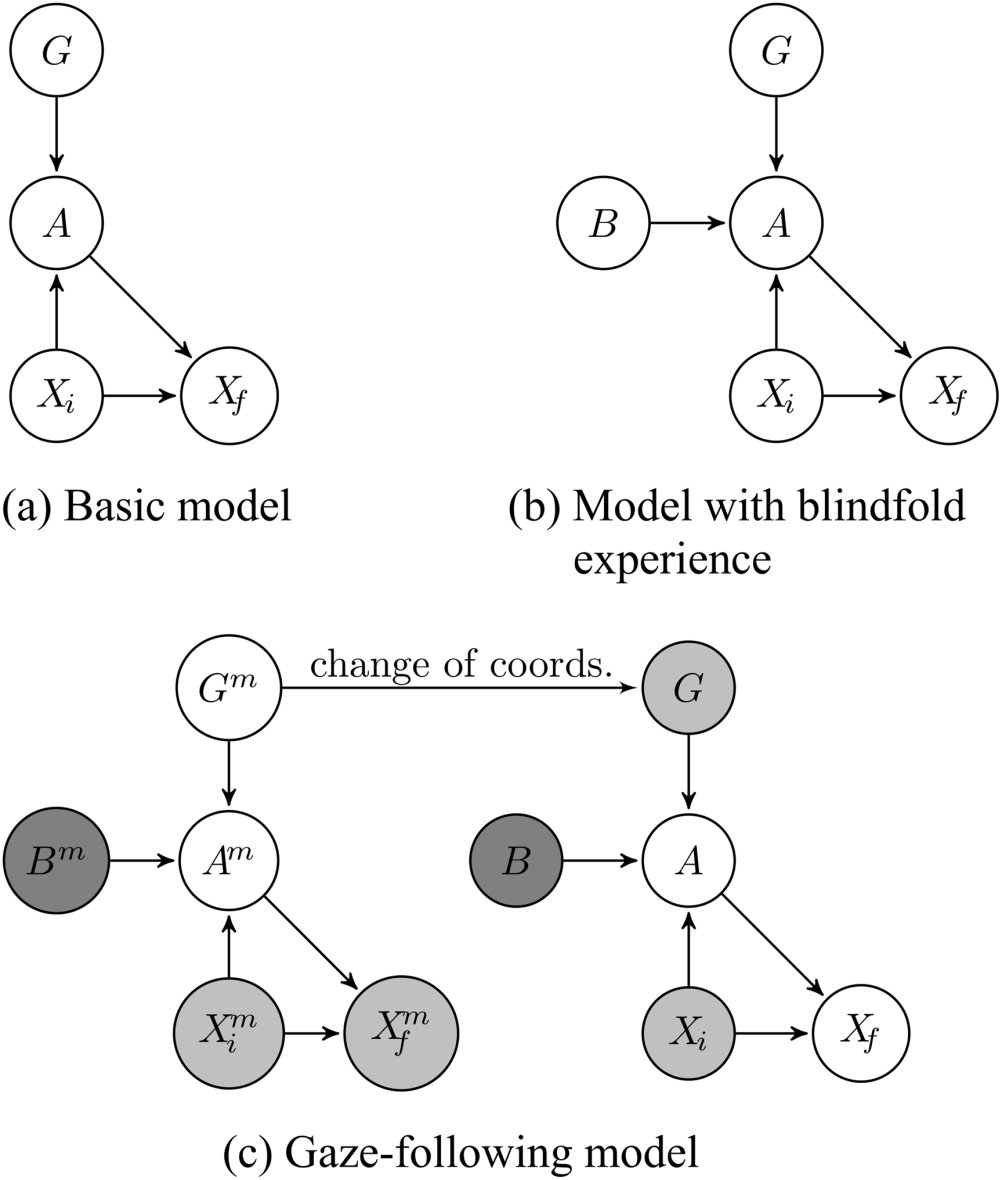
Graphical models for gaze following. (a) The graphical model shows the probabilistic dependencies between different random variables in the model: *G* = goal, *A* = action, *X*_*i*_ = current state, and *X*_*f*_ = final state. The model captures how actions depend on goals and states, and how the state changes as a consequence of executing an action; (b) incorporates the influence of blindfold self-experience on the model using the random variable B; (c) shows the combined graphical models, one for the agent and a copy for the mentor (superscript *m*), for following the gaze of a mentor. Shaded variables denote observed variables. The darker shading indicates that *B* is an observed discrete variable, while the rest of the nodes are continuous.

Our hypothesis is that humans use a goal-directed mechanism for planning motor acts. A goal, provided by either an internal desire or external stimulus, together with the current state, determines what the action should be–this is called a “policy.” An action executed in a given state in turn determines the next state (probabilistically). We represent these dependencies using the graphical model shown in Fig 1a where *G* is the goal, *A* is the action, *X*_*i*_ is the current (or initial) state, and *X*_*f*_ is the next (or final) state. Throughout this paper, we use upper case letters to denote random variables and lower case letters to denote specific values of the variables.

Consider our two example scenarios. In the context of gaze following, the goal is a desired fixation location, the action is a vector of motor commands to move the head, and the state represents head position and orientation. We discuss the model assuming movement of the head but the model can be generalized to embody movement of the eyes or movement of both the head and eyes to achieve a spatial target. In this case, the random variables (states, actions, goals) are continuous-valued and we define the relationships between them using (learned) *probabilistic functions*. In the case of our example of actions on objects, we assume the states represent discrete locations on a table, actions comprise of high-level commands such as pick and push, and the goal is a desired (discrete) location on the table. We use this example to illustrate the discrete formulation of the model in which the relationships between the discrete-valued random variables are captured by (learned) probability tables.

### Computational Model

#### Case I: Continuous-Valued Random Variables

We begin by showing how an agent can learn the mapping between continuous-valued goals, states, and actions which can later be used for planning and intent inference. The agent first learns a transition model *f*, (e.g., through self-exploration using its own body movements, which [11] dubbed “body babbling”). This translates an initial state, *x*_*i*_, and an action, *a*, to a final state, *x*_*f*_:

$$\begin{array}{c} x_{f}=f\left(x_{i},a\right)+\nu ,\;\nu ∼N\left(0,\Sigma_{\nu }\right), \end{array}$$

where N(*μ*, Σ) signifies the normal distribution with mean *μ* and covariance matrix Σ.

To make the exposition concrete, we focus here on the specific case of modeling gaze following, although the framework is general enough to apply to other types of behaviors. In gaze following, the initial and final state correspond to head poses while the action corresponds to motor commands to move the head. The learned transition model can in turn be used to learn a “policy” function, *π*, which maps an initial state and a goal location *g* to an appropriate action:

$$\begin{array}{c} a=\pi \left(g,x_{i}\right)+\upsilon ,\;\upsilon ∼N\left(0,\Sigma_{\upsilon }\right) \end{array}$$

This equation essentially determines the rotation required to turn the head from its current position to face the goal.

We use two separate Gaussian processes (GPs) [12], *GP*_*f*_ and *GP*_*π*_, to learn the two nonlinear functions *f* and *π* in the equations above. GPs are commonly used in machine learning to infer a function *h* from noisy observations *y*_*i*_:

$$\begin{array}{c} y_{i}=h\left(x_{i}\right)+\nu ,\;\nu ∼N\left(0,\sigma_{\nu }^{2}\right) \end{array}$$

GPs are both flexible and robust. First, they are nonparametric so they do not limit the set of functions that *h* can be chosen from. Second, they estimate a probability distribution over functions instead of choosing a single most-likely function. This allows all plausible functions to be incorporated into the estimation process and reduces model bias. For these reasons, GPs are effective with small numbers of training samples, increasing their biological plausibility. We now show how these GPs can be used for planning, goal inference, and gaze-following.

#### Goal-directed Planning

GPs are trained via supervised learning: given a training dataset with noisy labels, the GP learns the functional mapping between the input data and the labels (output). The training data could be obtained, for instance, through a reinforcement-based paradigm that combines exploration of the goal-action-state space for training the transition GP with selection of data from successful trials for training the policy GP (see [3]).

After training, it is simple for the agent to fixate on a goal location. We assume that the agent knows its current state *x*_*i*_ and the goal *g*. Given these, the agent uses its learned distribution over functions, *GP*_*π*_, to compute a probability distribution over actions, *P*(*a*) = N(*μ*_*a*_, Σ_*a*_). This distribution can then be passed through *GP*_*f*_ to estimate the probability of the resulting state *P*(*x*_*f*_) ≈ N(*μ*_*x*_*f*__, Σ_*x*_*f*__). Thus, our model provides both a prediction of the final position and an estimate of the uncertainty in the prediction. We define the combined inference process *P*(*X*_*f*_∣*X*_*i*_ = *x*_*i*_, *G* = *g*), as *Forward Inference*.

#### Goal Inference

One can also infer the goal of an observed action (e.g., head movement), given starting and ending states (e.g., head poses), *x*_*i*_ and *x*_*f*_, respectively. To accomplish this, the agent must be able to recover the inputs to each GP, given the outputs. Fortunately, results from [13] allow us to estimate a distribution over the inputs given the outputs. We follow the technique in [13] to infer a distribution over actions given *x*_*i*_ and *x*_*f*_ and then use this to estimate a distribution over goals. We define the inference process *P*(*G*∣*X*_*i*_ = *x*_*i*_, *X*_*f*_ = *x*_*f*_) as *Reverse Inference*.

#### Gaze Following

Gaze following of a mentor using the above model is accomplished by invoking Meltzoff’s “Like-Me” hypothesis. The agent learns a model of its own head movements and assumes that a mentor uses this same (or similar) model. As suggested by the graphical model in Fig 1c, the agent observes the starting and ending states (head poses) of a mentor and then infers the goal location indicated by the mentor, *g* ^*m*^, by using a copy of its own learned model and inferring what it would be looking at if it were in the mentor’s position. After inferring the mentor’s goal, the agent transforms that goal into its own coordinate frame and then infers how to fixate that goal. For this paper, we assume the agent has acquired the ability to transform between coordinate frames through prior experience. We acknowledge that the problem of transformation between the agent and mentor coordinate frames can be challenging in more complex tasks, and we refer the reader to relevant work in this area for further information [14–16].

#### Modeling Blindfold Experiments in Human Infants

We first tested the continuous-valued version of the model on a gaze-following task previously used in experiments in human infants [6]. One set of experiments showed that 14- and 18-month olds do not follow the gaze of an adult who is wearing a blindfold, although they follow gaze if the adult wears the same band as a headband. This suggests that these children did not follow gaze because they are able to take into account the consequences of wearing a blindfold (i.e., occlusion) and unlike the 12-month olds, make the inference that the adult is not looking at an object. This observation is closely related to Meltzoff’s “Like me” hypothesis [7]. In particular, self-experience with own eye closure and occluders may influence gaze-following behavior. To test this hypothesis, Meltzoff and Brooks provided one group of 12-month olds with self-experience with an opaque blindfold while two other groups either had no self-experience or had self-experience with a windowed blindfold. On seeing an adult with a blindfold turn towards an object, most of the children with self-experience with blindfolds did not turn to the object while the other two groups did [6]. This highlights self-experience as a learning mechanism that can used to interpret the behaviors of other agents.

To model these results, we incorporate a binary random variable *B* ∈ {0,1} in the graphical model, as shown Fig 1b, which denotes whether or not a blindfold is being worn, and allows the agent to learn the effects of being blindfolded. Our model learns a new Gaussian process ($GP_{\pi }^{B}$ in place of *GP*_*π*_) which is used when the agent is blindfolded. When the opaque blindfold is in place, regardless of the current value of the goal state, no action leads to that goal location being fixated. Goals in this case are not causally linked to states (head poses) or actions. The agent can learn this and then apply this knowledge to a mentor agent to infer that the mentor does not have a goal when blindfolded (i.e., the inferred distribution over goals approximates a uniformly random distribution). However, if this alternate Gaussian process is not learned, the agent does not know the consequences of wearing a blindfold and follows the mentor’s head movement even if the mentor is blindfolded. Fig 1c shows the combined graphical model for following the gaze of the mentor, based on combining a model for the agent and a copy for the mentor.

### Case II: Discrete-Valued Random Variables

When the random variables for states, actions, and goals are discrete-valued, the model can be expressed in terms of conditional probability tables for the transition probabilities *P*(*X*_*f*_∣*X*_*i*_, *A*) and the “policy” *P*(*A*∣*G*, *X*).

**Fig 2:**
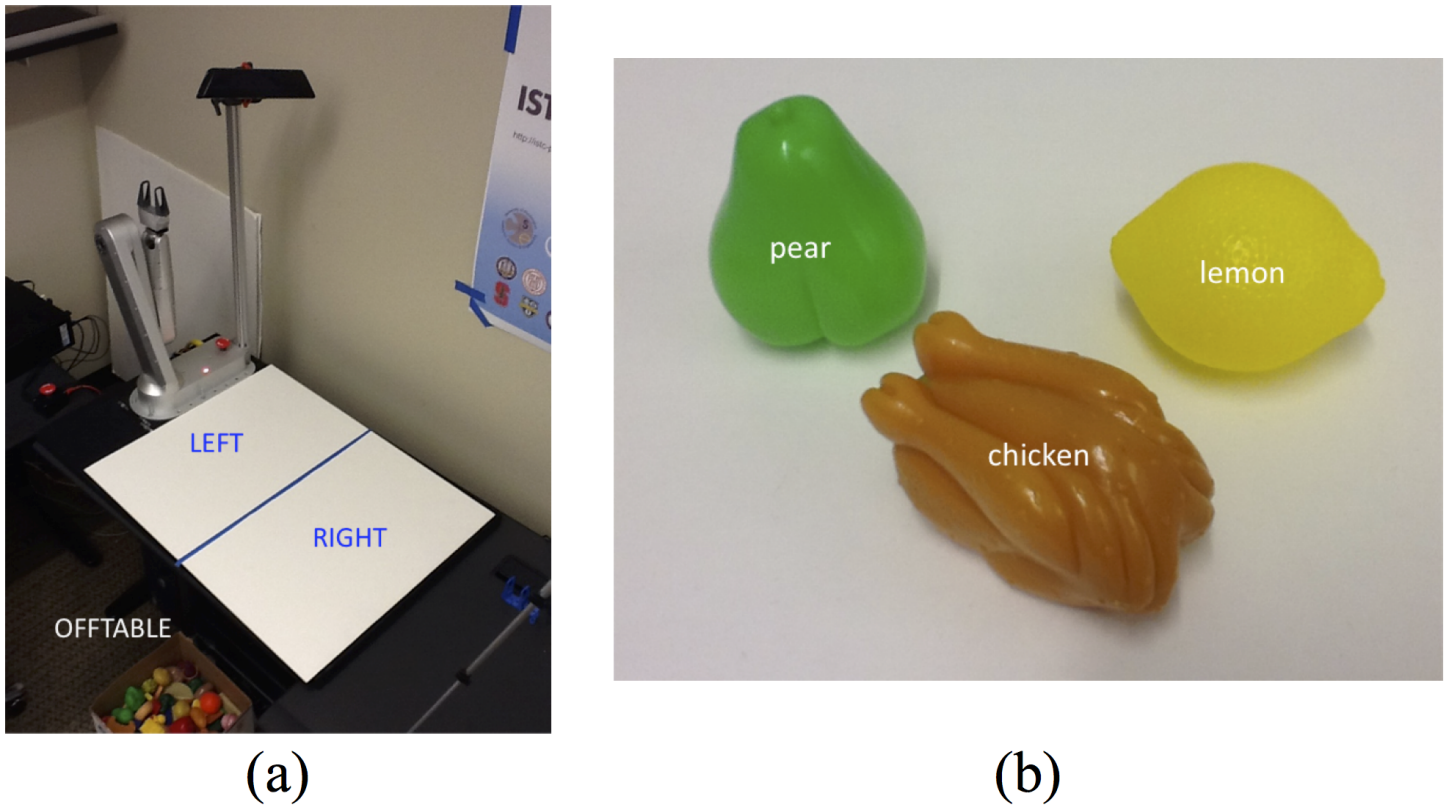
Robotic tabletop organization task setup. (a) The robot is located on the left side of the work area and the Kinect looks down from the left side from the robot perspective. The three predefined areas that distinguish object states are notated. (b) Toy tabletop objects.

For concreteness, we describe this case in the context of a simple tabletop task involving a set of small objects on a tabletop which can be moved around by a human or a robotic arm as shown in Fig 2. The discretized position of an object defines its *state* and a *goal* corresponds to the object reaching a particular state. The robotic arm can manipulate the state of any object using a set of *actions*. We define Ω^*X*^ to be the set of discrete states in the environment, Ω^*A*^ to be the set of all possible actions available to the robot (these can be different from the possible actions of the human demonstrator) and Ω^*G*^ to be the set of possible goal states. We assume all three sets are finite.

**Fig 3:**
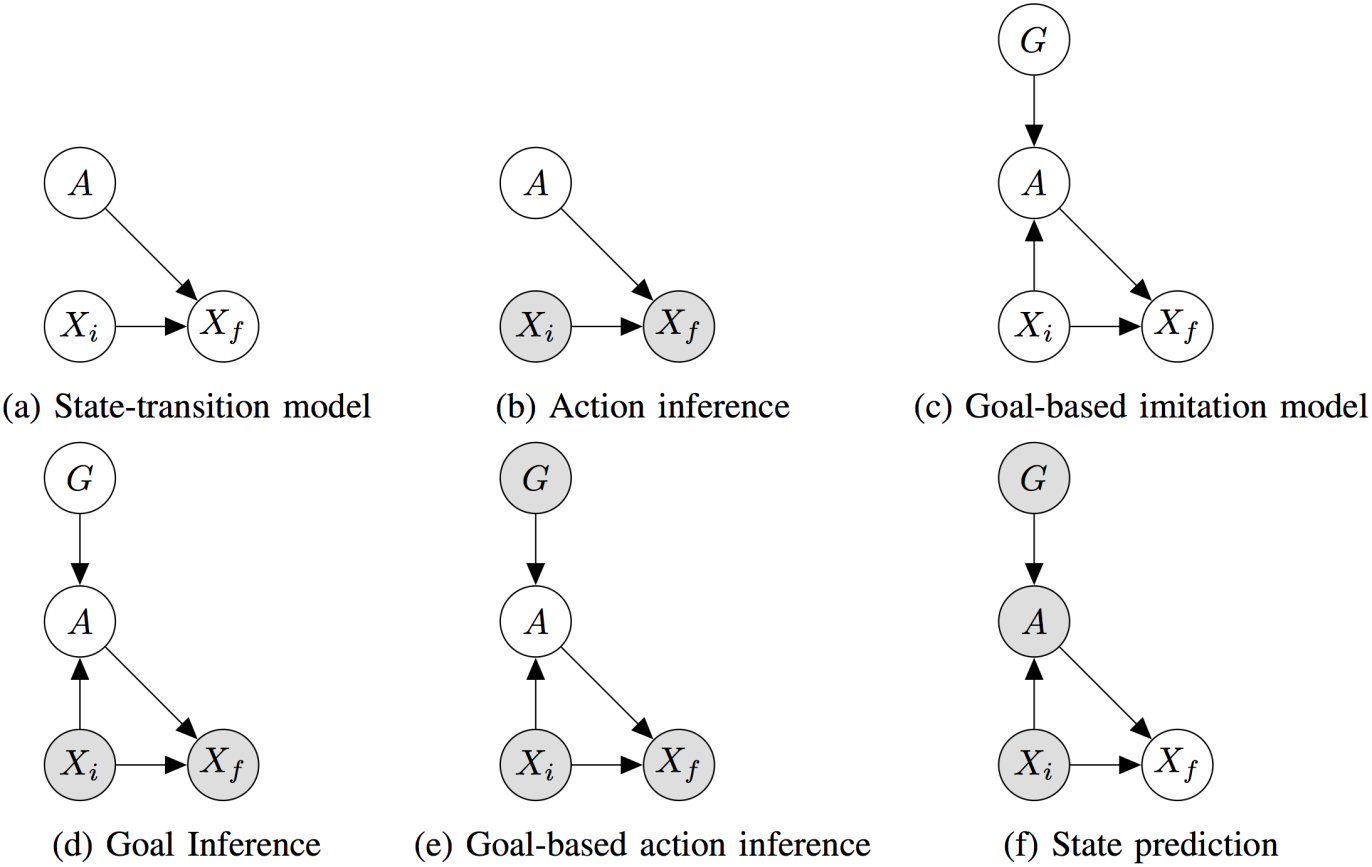
Graphical models for robotic goal-based imitation. (a) through (f) illustrate the use of graphical models for learning state-transitions, action inference, goal inference, goal-based imitation, and state prediction. Shaded nodes denote observed variables.

Each goal *g* ∈ Ω^*G*^ represents an abstract task which can be achieved using one or more actions in Ω^*A*^. For example, a goal can be moving an object to location A regardless of its starting location. The object could be picked and placed at A, pushed to A, or transported using some other action, but the goal remains the same. The dynamics of the action and its effect are modeled as a Markov state-transition model (Fig 3a); when the robot in a state *x*_*i*_ executes an action *a*_*i*_, it enters a state *x*_*f*_ with the transition probability *P*(*X*_*f*_ = *x*_*f*_∣*X*_*i*_ = *x*_*i*_, *A* = *a*_*i*_).

#### Learning Through Self-Experience

We assume that the transition probability distribution is initially unknown to the robot and must be learned through exploration, similar to the manner in which infants explore the consequences of their actions through exploration and “body babbling” [11, 17]. The robot collects training data tuples (*x*, *a*, *x*′) for the graphical model in Fig 3a by executing a random action *a* from a random initial state *x* and observing a resulting final state *x*′ multiple times. Given this training data, maximum likelihood parameter estimation is used to learn parameters for the transition probability distribution *P*(*X*_*f*_∣*X*_*i*_, *A*).

#### Goals and Goal Inference

After the transition parameters are learned, a goal-based graphical model, 𝓖, is created by augmenting the initial model in Fig 3a with a new node *G* as shown in Fig 3c. The robot then engages in goal-based exploration in which a goal state *g* is chosen at random and for any given initial state *X*_*i*_ = *x*_*i*_, the robot performs Bayesian inference to infer *P*(*A*∣*X*_*i*_ = *x*_*i*_, *X*_*f*_ = *g*). It samples an action A* from this distribution and executes this action. If the goal *g* is reached, it increases the “policy” probability *P*(*A* = A*∣*G* = *g*, *X*_*i*_ = *x*_*i*_) by a small amount, and decreases *P*(*A* = *A*′∣*G* = *g*, *X*_*i*_ = *x*_*i*_) for all other actions *A*′. An alternate approach, which we follow here, is to directly set *P*(*A*∣*G* = *g*, *X*_*i*_ = *x*_*i*_) = *P*(*A*∣*X*_*i*_ = *x*_*i*_, *X*_*f*_ = *g*). In this case, the policy is based purely on the plan inferred from a learned transition model without verification that a goal can be reached, so the policy’s accuracy will depend on the accuracy of the transition model. For the simple tabletop experiment we illustrate here, we adopt the latter approach given that accurate transition models can be learned for the small state and action space.

For goal inference, the robot observes object states $x_{i}^{h}$ and $x_{f}^{h}$ (e.g., object locations) from a human demonstration. By invoking the “Like me” hypothesis, the robot uses its goal-based graphical model to compute the posterior distribution over goals *G* given $x_{i}^{h}$, and $x_{f}^{h}$, as depicted in Fig 3d (note that the variable *A* is marginalized out during goal inference).

#### Goal-Based Imitation and Action Selection

Goal-based imitation is implemented as a two-stage process: (i) the robot infers the likely goal of the human demonstration using the goal-based graphical model described above, and (ii) either executes the action most likely to achieve this goal, or seeks human assistance if the goal is found to be unlikely to be achieved. Specifically, the robot senses the current state $x_{i}^{\prime }$ using its sensors and infers the human’s goal *g*_*MAP*_ by taking the mode of the posterior distribution of *G* from the goal-inference step. It then computes the posterior over actions *A* as shown in Fig 3e and selects the maximum a posteriori action *a*_*MAP*_. Since *a*_*MAP*_ is not guaranteed to succeed, the robot predicts the probability of reaching the most probable final state $x_{f}^{\prime }$ using *a*_*MAP*_ by computing the posterior probability of *X*_*f*_ as shown in Fig 3f. If this probability of reaching the desired state is above a prespecified threshold, *τ*, the robot executes *a*_*MAP*_, otherwise it executes the “Ask human” action to request human assistance.

### Robot

We use the Gambit robot arm-and-gripper (Fig 2a) designed at Intel Labs Seattle. Gambit is well-suited to tabletop manipulation tasks with small objects and has previously been shown to perform well in tasks with humans in the loop [18].

#### Sensing

For sensing the current state of objects on the table, for example, during human demonstrations, we use the Microsoft Kinect RGBD camera. The Kinect is mounted on the base frame of Gambit and looks down on the table surface. The robot takes as input the stream of RGB and depth images from the Kinect and first segments out the background and the hand of the human holding the small objects. The remaining pixels are then used to determine the state of objects on the table using a simple heuristic based on centroids. We define three discrete areas that are used for defining the state of the objects as shown in Fig 2a–(1) “LEFT” signifying that the object is on the left side of the blueline on the table; (2) “RIGHT” denoting that the object is on the right side of the blueline; and (3) “OFFTABLE” signifying that the object is not in the tabletop work area.

#### Robot Action

We assume that the robot possesses a fixed set of six high-level actions for manipulating objects: place LEFT (PlaceL), place RIGHT (PlaceR), place OFFTABLE (PlaceOt), push to LEFT (pUshL), push to RIGHT (pUshR), and push OFFTABLE (pUshOt). For the “place” actions, the robot first attempts to pick up the object by moving its end effector above the centroid of the object and rotating the gripper to align itself perpendicular to the major axis of the object. If the robot successfully picked up the object, it places the object down at the location (LEFT, RIGHT, or OFFTABLE) indicated by the place command. For the “push” actions, the robot first positions its end effector behind the object based on its centroid and direction of the push. For pUshL and pUshR, the gripper yaw angle is rotated perpendicular to the major axis of the table, while for pUshOt, it is rotated parallel to the major axis of the table. This ensures that object contact area is maximized to reduce the chance of the object slipping while pushing. The robot pushes the object until it changes state (or the inverse kinematics solver fails to find a possible solution).

## Results

### Gaze Following: Model Simulations

To test our model, we randomly sample goal positions and then compute the action required to fixate on this goal. We add Gaussian noise to this action, compute the resulting gaze vector if this action were taken, and add Gaussian noise to this gaze vector. This method is equivalent to training the model with rejection sampling wherein the agent rejects all samples that do not result in successful fixation on the goal position. The Gaussian processes are trained on this randomly generated data and then tested on separate test data. The default reference frame for both agent and mentor is at the origin gazing along the x-axis. Each agent has their own reference frame and we assume that we know the transformation from the mentor’s reference frame to the agent’s. This transformation is not learned by our model but we believe that this is a minor assumption, especially since we already assume the agent can observe the mentor’s position and head pose.

**Fig 4:**
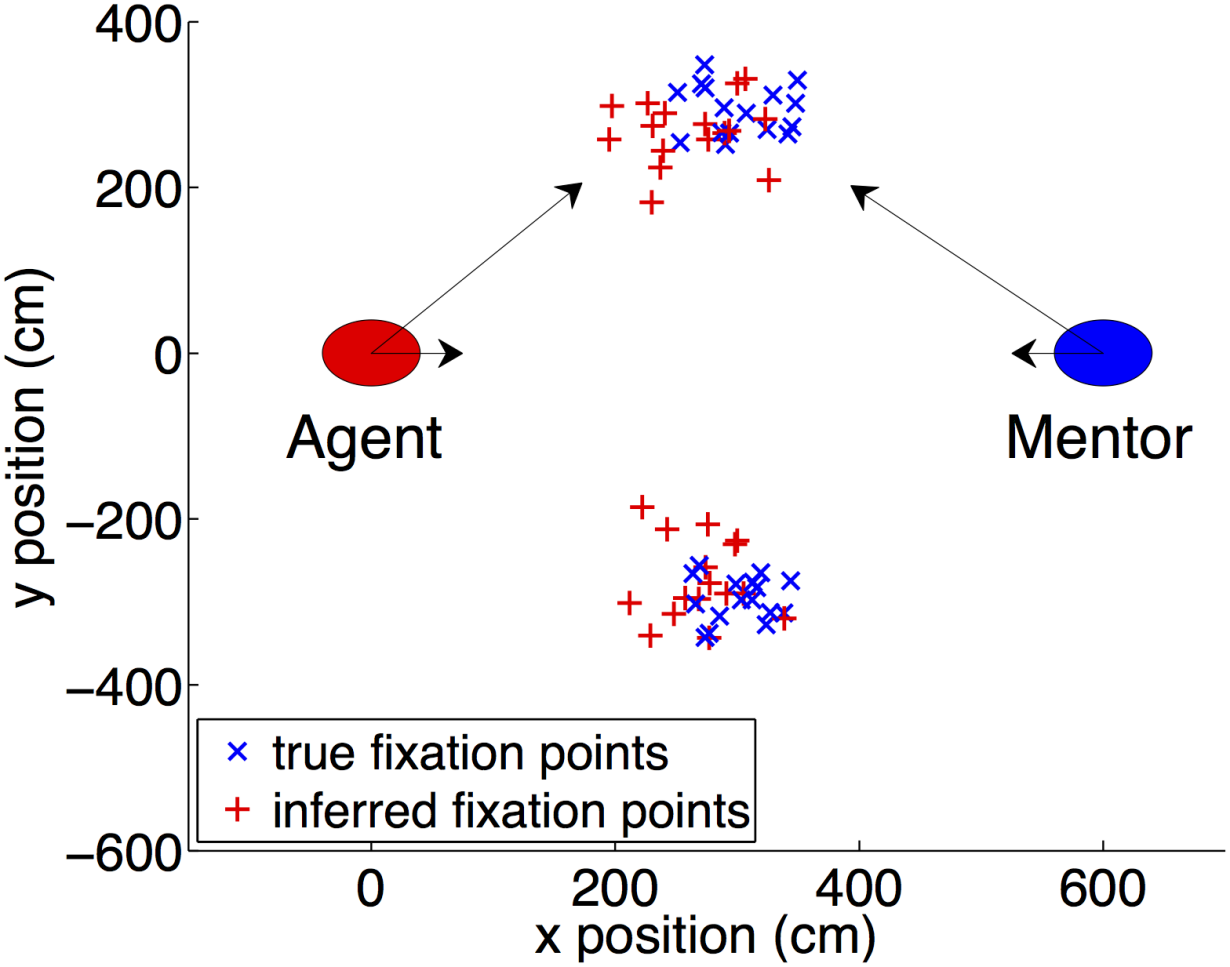
Gaze following results. The agent and mentor face each other in a 2D simulated environment. Goal positions inferred by the model from mentor observations are shown in red next to true goal positions (blue) for sampled goal positions both to the left and to the right of the agent. Black arrows represent the initial and final gaze vectors of the agent and mentor for one of these test data points. In this simple example, the goal locations were equidistant from the agent and mentor but the model readily generalizes to other cases as well.

The mentor and agent are positioned as shown in Fig 4. Goal locations for the training data were generated uniformly at random from the area between the agent and the mentor (within the rectangle formed by *x* in [100, 500] and y in [−500,500], where the agent is at (0,0) and the mentor is at (600,0)). We used Gaussian noise with standard deviation of 3 degrees for angles and a standard deviation of 10 cm for locations and distances. For reverse inference, the prior goal state *P*(*G*) is a Gaussian centered halfway between the two agents along the x-axis. While this prior is quite weak, a single observation of (*x*_*i*_, *x*_*f*_) is insufficient to overcome the prior in reverse inference. Instead, we use a single observation of *x*_*i*_ and five observations of *x*_*f*_ to get an accurate estimate of the goal distribution *P*(*G*∣*x*_*i*_, *x*_*f*_). More precisely, we run reverse inference with the observed values $\left(x_{i},x_{f}^{\left(1\right)}\right)$ to compute $P(G|x_{i},x_{f}^{\left(1\right)})$, and then use this as the prior for a second run of reverse inference to compute $P(G|x_{i},x_{f}^{\left(1:2\right)})$. We repeat this five times to compute $P(G|x_{i},x_{f}^{\left(1:5\right)})$. We believe such an inference process could be executed within the short amount of time taken for gaze following.

#### Gaze Following: Model Performance

We found that the model learns accurate transition and policy functions from small amounts of noisy training data (*n* = 200 data points in our accuracy tests). The nonparametric nature of Gaussian processes ensured very little customization was required. The model runs in sub-minute times for the dimensionality we are using, though additional approximations may be necessary to scale well to high dimensions. We also found that the Gaussian approximations we are making have little effect because the data is generally unimodal and close to symmetric.

**Fig 5:**
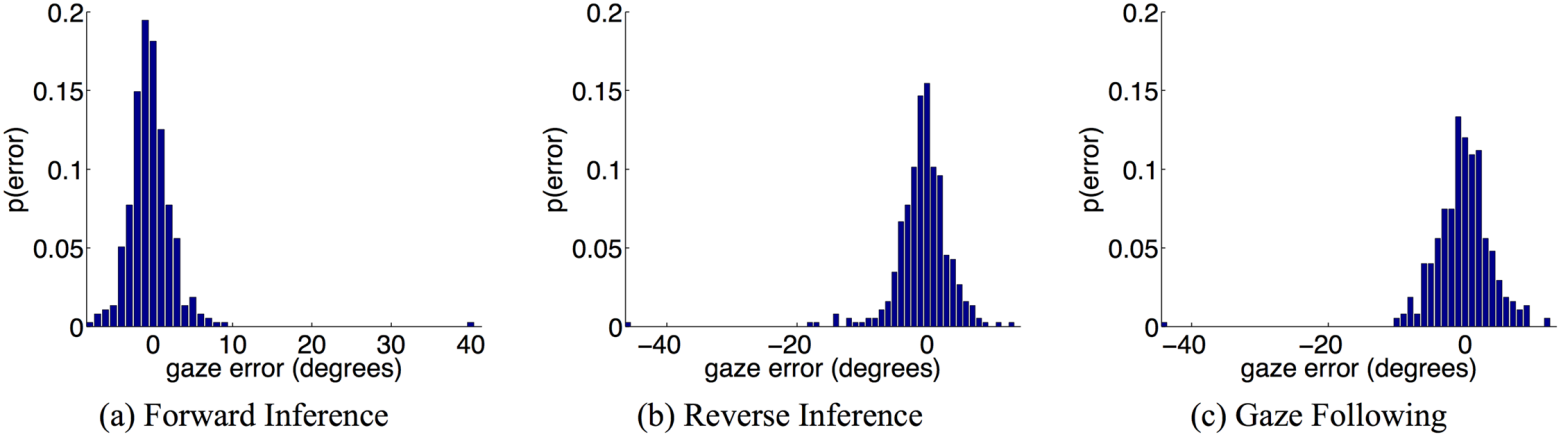
Accuracy of goal inference and gaze following. Histogram plots showing the probability of an error (in degrees) between the inferred and the true gaze vector–the gaze vectors in the final state *X*_*f*_ were used for (a) and (c), and the gaze vectors in the the goal state *G* were used for (b). These probabilities were estimated from 375 test points spread uniformly over the test region. Note how the accuracy gracefully decays as more complicated inference is performed ((a) is the simplest, while (c) is the most complex).

Figs 4 and 5 show performance results for the model as it performs forward inference, reverse inference, and gaze following (combined reverse and forward inference). The model is robust to noise and is able to provide accurate gaze following results even though additional levels of uncertainty are introduced by the second level of inference.

#### Blindfold Self-Experience Task

In order to test the cognitive plausibility of our model, we recreated the experiments from [6], where infants’ self-experience with a blindfold affects whether or not they follow the gaze of blindfolded adults (see Fig 1c). We trained 60 separate agents with our model on randomly generated training data. Similar to the infant experiments, one third of these agents were given additional experience with a blindfold wherein they train an additional *GP*_*π*_ for their model so that they now have *GP*_*π*_ and $GP_{\pi }^{B}$, where *GP*_*π*_ is the original GP of the model and $GP_{\pi }^{B}$ is the GP with blindfold experience. The agents with the blindfold experience learn the consequences of wearing an opaque blindfold in $GP_{\pi }^{B}$. The other 40 agents were trained normally although one group is the baseline group and the other is the windowed blindfold group. In our simulations, the windowed blindfold group experiences the same training data as the no-blindfold case since, in both cases, the overall result is that the target is visible to the agent. So these two groups will be identical except for noise.

Each agent is presented with 4 trials where it observes a mentor make a head turn to face either 45 degrees to the left or 45 degrees to the right (plus noise). Trials are scored as +1 if the agent turns its head at least 30 degrees in the direction of the correct target and −1 if it turns its head at least 30 degrees in the direction of the wrong target. The agents with no blindfold experience use the basic model (which contains no blindfold knowledge) and thus assume that the mentor is fixating on an object to the left or to the right. Those with blindfold experience observe that the mentor is wearing a blindfold and use their learned $GP_{\pi }^{B}$ for the reverse inference (applying their model to the mentor). For this group, we expect the agent to have learned through self-experience that when blindfolded, there is no correlation between a head movement and any particular goal position. The blindfold-experienced agent then uses *GP*_*π*_ for the forward inference (because the agent is not wearing a blind-fold). In order to simulate infants with little experience with blindfolds, we provided the agents in this experiment with very little training data (*n* = 15). If we use more training data, the agents perform almost perfectly in this task.

**Fig 6:**
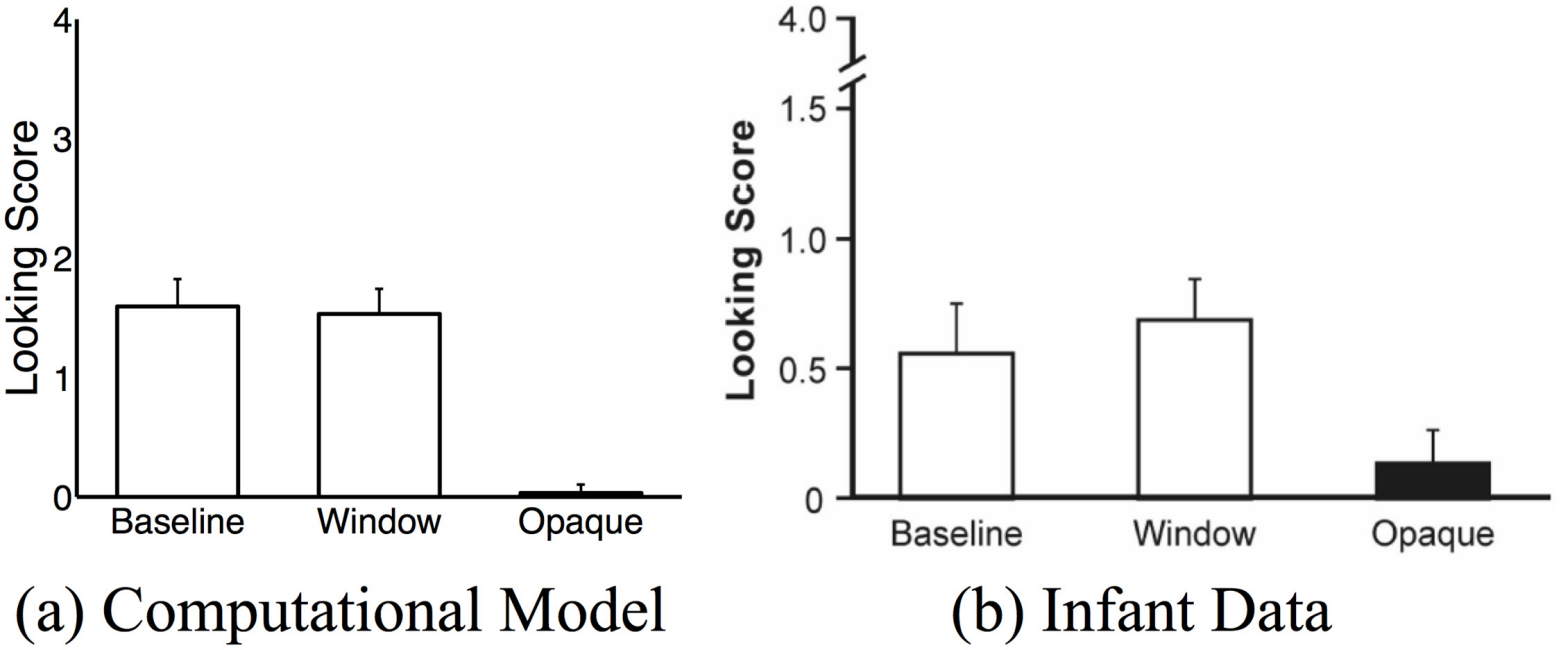
Comparison of the model to infant data from [6]. Error bars represent standard error of means.

Results are shown in Fig 6 and match the pattern of results in Meltzoff and Brooks (reproduced in Fig 6a for comparison). This suggests that gaze following involves understanding the underlying intention or goal of the mentor, and that self-experience plays a major role in learning the consequences of intentions and related actions.

### Robotic Tabletop Manipulation Task

To illustrate the discrete-valued version of the model, we used a tabletop manipulation with three toy objects of different shapes: a “pear,” a “lemon,” and a miniature “broiled chicken.” These objects could be in one of three different states: LEFT, RIGHT, and OFFTABLE, as defined in the previous section. The robot’s aim is to learn the consequences of actions on these objects, infer the goals of human actions, and imitate the actions of humans manipulating these objects on the table.

**Fig 7:**
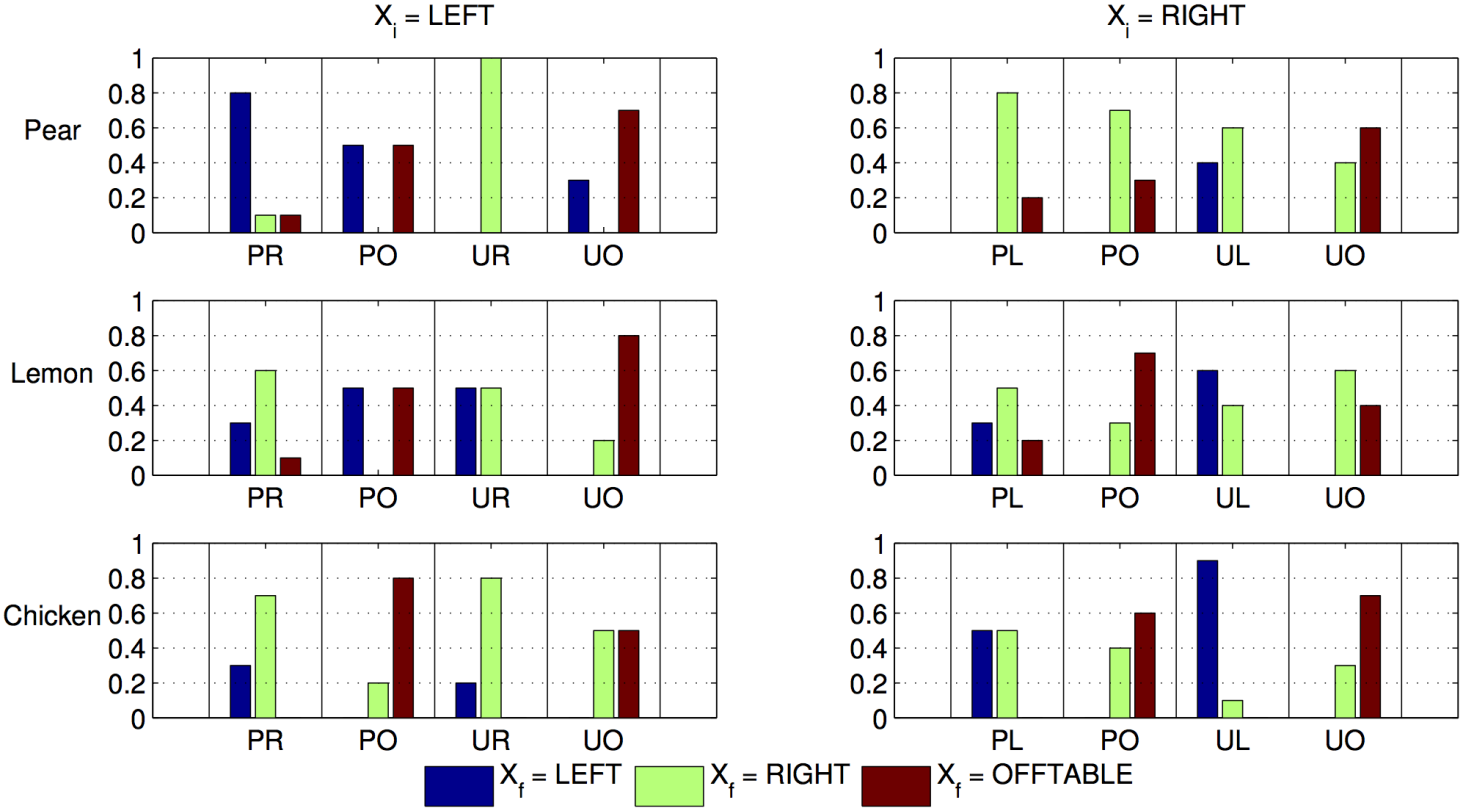
Robotic task: Learned transition model. Each row represents different types of objects, and each column represents the different initial states *X*_*i*_. The colors of bars represent different final states *X*_*f*_. The y-axis represents the range of probabilities and the x-axis represents the six different manipulation actions available to the robot (PL = place LEFT, PR = place RIGHT, PO = place OFFTABLE, UL = pUsh LEFT, UR = pUsh RIGHT, UO = pUsh OFFTABLE). We do not show actions that cause self-transitions given an initial state.

Fig 7 shows the learned transition probabilities for manipulating these objects based on the robot’s self-discovery phase. The transition models are learned by performing 10 trials for each initial state and action pair (*x*_*i*_, *a*) for each object type. We deliberately used a small number of trials to test whether the method could cope with less training data and more uncertainty in the transition model.

Since the state space is small, we are able to enumerate and test all of the interesting and possible human demonstrations. By interesting, we mean that the state changes after action execution (for example, from RIGHT to OFFTABLE) and the initial state is not OFFTABLE. There are in total four interesting state changes for each object that can be demonstrated by a human: LEFT to RIGHT, LEFT to OFFTABLE, RIGHT to LEFT, and RIGHT to OFFTABLE. Note that our current implementation does not allow the robot to pick up objects that are located OFFTABLE

**Fig 8:**
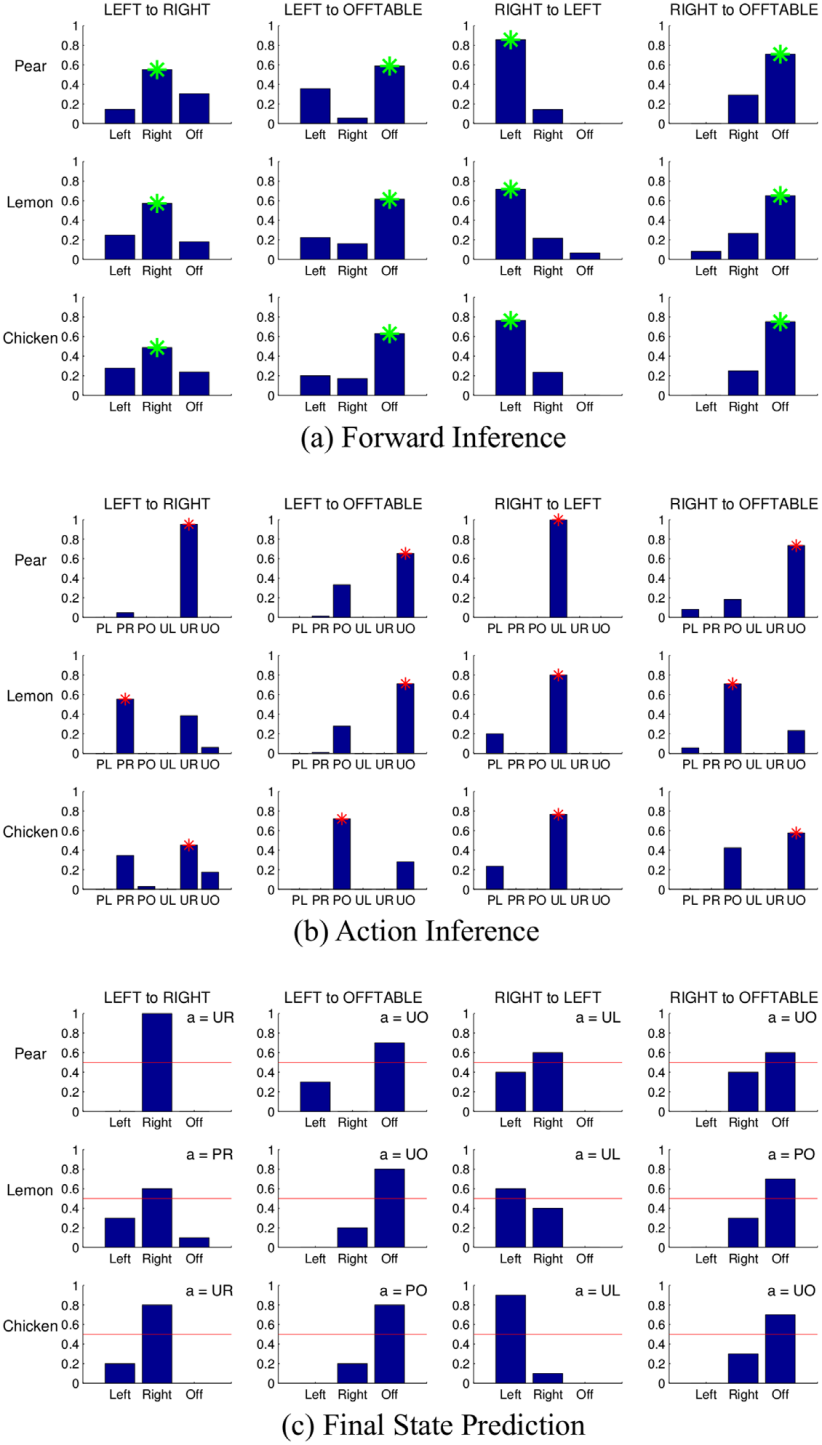
Robotic task: Results. (a) **Most likely goals**: Initial and final states are at the top of each column. The height of the bar represents the posterior probability of each goal state, with the true goal state marked by an asterisk. (b) **Inferring actions**: For each initial and desired final state, the plots show the posterior probability of each of the six actions, with the MAP action indicated by an asterisk. (c) **Predicting final state**: The plots show the posterior probability of reaching the desired final state, given the initial state and the corresponding MAP action shown in (b). The red bar marks 0.5, the threshold below which the robot asks for human help in the Interactive Goal-Based mode.

Fig 8a shows the inferred goals given all possible interesting state changes, using the graphical model in Fig 3d. For all cases, our model correctly infers the intended goal state of the human. Fig 8b shows the maximum a posteriori probability (MAP) action for a given initial state and goal. Our model correctly identifies whether a “place” or a “push” action is better, given the dynamics of the object as encoded in the learned probabilistic model for the object. Note that the two most preferred actions are always push or place actions in the correct direction.

Finally, Fig 8c shows the predicted state distribution given an action and an initial state. The robot calculates the posterior probability of getting to the desired state, and executes the action if this probability is above a predetermined threshold (%50). Otherwise, it asks the human collabo For example, the predicted output of moving a pear from RIGHT to LEFT (1st row of 3rd column in Fig 8c) using the pushing left action is lower than %50, and therefore the robot will ask request the human help.

Table 1 compares “trajectory-based” imitation of the human demonstration with our proposed goal-based approach. The trajectory-based approach simply mimics the human action without considering the goal or uncertainty, i.e., it executes a place action if the human executes a place, and a push action if the human executes a push. The goal-based approach on the other hand recognizes the goal and uses the best action it has available to achieve the goal.

**Table 1:** Comparison of approaches.

	Trajectory	Goal-Based	Interactive Goal-Based
Pick & Place Demonstration			
Pear	0.2250	0.6750	***0.8250***
Lemon	0.5250	**0.6750**	**0.6750**
Chicken	0.6500	**0.8000**	**0.8000**
Push Demonstration			
Pear	0.6750	0.6750	***0.7250***
Lemon	0.5750	**0.6750**	**0.6750**
Chicken	0.7250	**0.8000**	**0.8000**

The hypothetical success rates of three different approaches to imitation are shown. The success rate shown in each cell is averaged over four interesting state changes mentioned in the main text. The boldfaced results indicate the row-wise highest success rates, and the cases where the robot’s request for human help improved success the rates are shown in italic.

Using our computed transition probabilities, we can calculate the hypothetical success rate of a purely trajectory-based approach. For our goal-based approach, we use the posterior distribution shown in Fig 8b. Finally, the “Interactive Goal-Based” mode assumes that the robot may ask a human for help, with a 100% success rate when the human executes the requested action. The third column in Table 1 shows what the performance would be if we require the robot to be 50% sure of reaching a desired state. We do not see perfect imitation results on the third column because the robot does not ask the human for help in every case. In some cases, the probability of success will surpass the confidence threshold, but the goal state may not be reached after the action is executed.

The results demonstrate the expected behavior of the goal-based method. The success rates in goal-based method (Table 1, second column) are identical across the different actions demonstrated (e.g. pick & place row vs. push row). In addition, the results demonstrate the advantage of a goal-based approach over purely trajectory-based imitation, and its potential as a human-robot collaboration mechanism.

## Contributions and Comparisons with Related Work

Our work makes contributions both to the fields of robotics and cognitive modeling. In robotics, our approach contributes to the growing number of efforts leveraging biological paradigms and developmental science to design new methods for social robotics and human-robot interaction [17, 19–30]. Specifically, our proposed framework for goal inference and imitation lays the foundation for a new approach to designing social robots as robots that learn internal models of their environment through self-experience and utilize these models for human intent recognition, skill acquisition from human observation, and human-robot collaboration. Although our results are based on proof-of-concept systems, the underlying Bayesian framework is general. We anticipate being able to progressively increase the sophistication of our models by leveraging the rapid advances being made in probabilistic reasoning, Bayesian modeling, and learning. As an illustrative example, the success of simultaneous localization and mapping (SLAM) algorithms in robotics [31] in the past decade can be largely attributed to the availability of efficient algorithms and computing platforms for probabilistic reasoning, within the broader context of probabilistic robotics [32].

Our approach to robotic imitation emphasizes the importance of goal inference in imitation learning, compared to traditional methods for programming-by-demonstration that have relied on following action trajectories. Previous approaches to robotic imitation that have also relied on high-level goals (e.g., [33, 34]) have not emphasized self-discovery of probabilistic models, a central tenet of the developmental approach proposed here for bootstrapping goal-based imitation. Other robotics work has focused on attempting to model continuous low-level goals [35]. Neither of these approaches adopt probabilistic models for human-robot interaction tasks. Approaches that do utilize Bayesian methods for goal inference [36, 37] and robotic imitation [38] have done so without the developmental science perspective that we bring to the problem.

In the context of cognitive modeling, our model can be regarded as a Bayesian instantiation of the “Like-Me” developmental hypothesis [7, 39]. It acknowledges a role for a certain type of mental simulation [8] as well as a learned “theory” [9, 10] of actions and their consequences using a probabilistic model learned from experience (see also [40]). The model is closely related to the goal-based imitation model of Verma and Rao [3, 17, 41] and the inverse planning model of Baker et al. [42]. Also related to our approach are models for planning based on probabilistic inference [3, 43–46].

Meltzoff’s “Like-Me” hypothesis has previously been applied in robotics to tackle the important problem of “whom to imitate” [47]: In this case, a robot first builds a self model, and then uses this self-model to discover self-other equivalences with other robots so as to distinguish an appropriate from an inappropriate “mentor” robot. Note that in this case as well as in our own work, the “mentor” agent is not necessarily an active teacher but is only being observed (cf. [48]). A recent developmental approach to robotic imitation [49] also focuses on tabletop manipulation tasks, with greater complexity than our experiments, but without the benefits of bootstrapping derived from the “Like-Me” hypothesis inherent in our framework.

## Summary and Conclusion

Our results suggest that the process of imitation-based learning can be bootstrapped by (i) learning a probabilistic model of the perceptual consequences of one’s own actions through self-experience, (ii) using this learned model to infer the goals of actions of others, and (iii) using the inferred goals to perform goal-based imitation. Such a strategy works even when the imitator’s actuators are different from the demonstrator’s. We first showed how such a model can emulate infant development of gaze following by implementing gaze following via goal inference in learned graphical models based on Gaussian processes. Using a table-top robotic system, we demonstrated that a robot can learn probabilistic models of actions on objects and utilize these models for inferring the intent of human actions on objects. Additionally, we showed that the approach facilitates human-robot collaboration by allowing the robot to predict the success probability of achieving a goal and to seek human help when success probability is low.

Our results point to a number of interesting open issues. For the studies in this paper, we used a simple exhaustive exploration strategy, but for larger state spaces, a more sophisticated approach based on reward functions (e.g., [50]) could be employed. Additionally, following the example of human infants, some form of directed self-exploration based on observing human mentors (e.g., [51]) may be desirable. The model assumes that states in the environment are known (corresponding to the case of MDPs or Markov decision processes)–an interesting direction for future work is extending the model to the more realistic case where only observations of states are available (partially observable MDPs or POMDPs) and where learning involves optimizing rewards. Finally, our current model involves inference over a single time-step, which simplifies the inference problem. We hope to explore the applicability of our model for multi-step inference problems in future work.

The approach we have presented lends itself naturally to generalization based on relational probabilistic models [52, 53] and hierarchical Bayesian representations [54]. Such models have the potential to significantly increase the scalability and applicability of our suggested approach to large-scale scenarios, besides facilitating transfer of learned skills across tasks and domains. We intend to investigate such relational models in future work.
